# Embodied Responses to Harmony and (Poly)Rhythm: An Integrated Ratio‐Based Perception‐Action Framework

**DOI:** 10.1111/nyas.70355

**Published:** 2026-08-08

**Authors:** Nicola Di Stefano, Edward Large, Charles Spence

**Affiliations:** ^1^ Institute of Cognitive Sciences and Technologies, National Research Council of Italy Rome Italy; ^2^ Music Dynamics Laboratory University of Connecticut Storrs Connecticut USA; ^3^ Crossmodal Research Laboratory University of Oxford Oxford UK

**Keywords:** consonance and dissonance, Farey tree, harmony perception, integer ratios, neural resonance theory, rhythm perception

## Abstract

Harmony and rhythm perception have traditionally been treated as separate perceptual phenomena in auditory cognitive science. Yet, both involve inherently oscillatory and periodic structures. Converging evidence suggests that harmonic and rhythmic patterns that can be described in terms of (approximate) low‐order integer relationships are often perceived differently from those characterized by more complex relations. In harmony, small integer frequency ratios (e.g., 2:1 for the octave, 3:2 for the perfect fifth) are related to perceived consonance and ease of auditory encoding. Similarly, rhythmic patterns whose component durations can be expressed by simple ratios (e.g., 2:1, 3:1) as well as polyrhythms based on simple ratios (e.g., 2:3, 3:4) are more readily detected, produced, and remembered than those based on more complex ratios. In this paper, we ask whether these perceptual and processing advantages reflect underlying shared mechanisms across harmonic and rhythmic domains. Furthermore, we explore whether and how these advantages extend into the motor domain. Grounding on neural resonance theory, we propose an integrated ratio‐based framework that links the perceptual salience of low‐order integer relationship in rhythm and harmony to enhanced sensorimotor synchronization.

## Graphical abstract

Harmony and rhythm perception are often studied separately, yet both reveal a shared sensitivity to low‐order integer ratios. In harmony, intervals like the octave (2:1) and fifth (3:2) predict consonance and ease of encoding. In rhythm, patterns with simple ratios (e.g., 2:1, 3:2) are easier to perceive, reproduce, and remember. Grounding on neural resonance theory, we propose an integrated perception‐action framework linking harmony, rhythm, and motor synchronization.

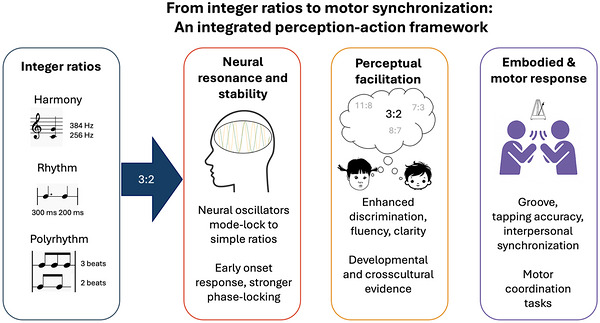

## 1 Introduction

Understanding how different combinations of sounds affect our perceptual experience of music has long been a central goal in the cognitive sciences of music. At its core, this endeavor seeks to uncover how the foundational elements of musical language combine into patterns that elicit significantly different perceptual effects on listeners. This question has often been translated in psychoacoustic terms in order to assess the effect of specific acoustic qualities of sounds, such as timbre and pitch contour, on listeners [1, 2]. Two key areas in which this question has been thoroughly explored are harmony/melody and rhythm, with research in each domain offering critical insights into how the auditory system processes sound structures, from isolated intervals/chords to complex musical excerpts [3, 4].

A central construct governing the combination of sounds in the harmonic and melodic domains is the distinction between consonance and dissonance [5, 6]. These terms refer to the perceptual qualities that emerge when two tones are played either simultaneously (harmony) or in succession (melody). Consonances are typically defined as perceptually smooth, stable, and pleasant, whereas dissonances are often associated with rough, tense, or unpleasant sensations [6, 7, 8, 9, 10]. This dichotomy has played a foundational role in the development of Western musical theory and composition, influencing how chords and intervals are combined into complex musical structures [11]. Since the first theorization by Pythagoras, consonance and dissonance have often been explained in terms of frequency ratios, with more consonant intervals being expressed by low‐order frequency ratios, and more dissonant by more complex ones [12]. For example, intervals such as the octave (2:1) or perfect fifth (3:2) are typically judged as highly consonant, while combinations of sounds whose frequency can be expressed by ratios such as 16:9 or 65:50 result in more dissonant intervals (though see [13] for an anti‐Pythagorean perspective).

Parallel to this, research has delved into rhythm perception by examining how listeners perceive and respond to rhythmic streams with different structures, from isochronous two‐beat patterns to the simultaneous combination of different streams, commonly referred to as polyrhythms [14, 15, 16]. Interestingly, a robust body of empirical research demonstrates that rhythmic sequences with temporally spaced events conforming to simple integer ratios (e.g., 2:3, with inter‐onset intervals of 200 and 300 ms) tend to be more easily perceived, reproduced, and remembered than those that are based on more complex relationships (e.g., 2.5:3, with inter‐onset intervals of 250 and 300 ms) [17, 18, 19, 20, 21].

Despite the fact that both harmonic intervals and (poly)rhythms are oscillatory and periodic phenomena in nature, research on harmonic and rhythmic perception has largely developed along separate lines. Yet, these two bodies of work converge on a strikingly similar conclusion: low‐order ratios play a central role in shaping perception in both the harmonic and rhythmic domains of music. Although ratios are conceptualized and implemented differently in each case—describing frequency relationships in harmony and patterns of event density over time in rhythm—this convergence raises intriguing questions. Specifically, what mechanisms underlie the apparent perceptual bias toward low‐order relationships in auditory processing? And, relatedly, in which ways do similar perceptual advantages extend to the motor domain?

In this review, we target these questions by integrating two traditionally separate domains of experimental research under a shared ratio‐based framework, namely, harmony (consonance) and (poly)rhythm perception. We argue that the perceptual saliency associated with consonant intervals and (poly)rhythms both stem from neural resonance theory (NRT) [22]. Furthermore, by integrating findings from psychoacoustics, neurophysiology, and behavioral studies, we aim to show how these processing mechanisms affect responses in the motor domain, in terms of movement coordination and synchronization in response to consonant harmonies or (poly)rhythms expressed by low‐order integer ratios.

The review is structured as follows. We begin by reviewing key findings from the literature on consonance and dissonance, showing that distinct perceptual and behavioral responses are consistently associated with harmonic intervals whose frequency relationships can be expressed as, or closely approximate, low‐integer ratios (i.e., consonances), as opposed to more complex ratios (i.e., dissonances), in both human and nonhuman listeners (Section 2). We then turn to the literature on rhythm perception, with a focus on those studies that have investigated how rhythmic ratios influence detection, reproduction, and preference in both human and nonhuman listeners (Section 3). Building on the evidence reviewed, we discuss how several perceptual attributes, such as fluency, discrimination, and clarity, characterize the processing of low‐order ratios in both domains (Section 4). Thereafter, we move on to integrate these findings, proposing a unified theoretical framework grounded in NRT, referring to mathematical notions such as the Farey tree sequence and nonlinear dynamical systems. We extend this framework beyond perceptual mechanisms to include motor responses, including tapping and locomotor–respiratory coupling (LRC), thus tracing a link between musical structure and embodied responses (Section 5). In the concluding section, we highlight some directions for future research in the field (Section 6).

## 2 Harmonic Consonance and Integer Ratios

The link between consonance and simple numerical relationships traces back to Pythagoras (sixth century B.C.E.), who is attributed with the first empirical observation on consonance, that is, that plucking two strings whose lengths are related by small integer ratios produces intervals that are perceived as harmonious. Pythagoras noted that the first four positive integers—1, 2, 3, and 4, collectively known as the *tetraktys*—could account for the most stable and pleasant‐sounding intervals in Western music: the octave (2:1), the perfect fifth (3:2), and the perfect fourth (4:3). In contrast, more complex ratios tend to yield increasingly dissonant or unstable percepts.

The idea that consonance reflects mathematical simplicity has informed musical theory for centuries. Thinkers such as Zarlino [23], Kepler [24], Rameau [25], and Euler [26] extended the Pythagorean framework, positing that harmony is fundamentally rooted in the simplicity of number. The reference to integer ratios persisted in Helmholtz's [27] influential psychoacoustic theory of consonance, which attributed dissonance to the phenomenon of beating and roughness—interference patterns generated by closely spaced partials in complex tones. Helmholtz's model claimed that simple frequency ratios minimize such interference, thereby preserving the perceptual smoothness associated with consonant intervals.

In the behavioral literature on auditory perception, several studies show that listeners typically exhibit different discrimination abilities for consonant as compared to dissonant stimuli [28, 29]. For example, the memory of a melodic interval based on low‐order frequency relationships (e.g., the perfect fifth, 3:2) is more stable than the memory of a melodic interval based on a more complex ratio (e.g., the tritone, 45:32) [30, 31, 32, 33]. In a recognition task involving harmonic intervals, Seror and Neill [34] found that pitch recognition was faster and more accurate when the probe tone was part of a consonant rather than a dissonant interval, particularly when the probe was the lower tone. These findings suggest that tone pairs that form simple frequency ratios (i.e., consonances) are perceived as different from those that form complex ratios (i.e., dissonances), and that such perceptual difference affects recognition, memorization, and discrimination abilities.

Neurophysiological evidence supports this discrimination ability, with consonant intervals evoking stronger and more temporally precise phase‐locking in the auditory nerve [35] and generating more synchronized cortical activity [36, 37]. The auditory cortex shows reduced adaptation to consonant intervals, suggesting they retain perceptual salience and resist habituation [38, 39]. In brainstem responses, consonant intervals yield more robust evoked potentials than dissonant ones [36], and the degree of dissonance correlates with a reduction in periodic neural activity [3, 40, 41, 42].

Research shows that the processing of consonance starts early in the human auditory cortex and that additional neural resources are needed to encode and discriminate dissonant as compared with consonant chords. Brattico et al. [43] and Tervaniemi et al. [44] found that consonant stimuli elicit less neural activity than dissonant ones, consistent with reduced processing effort. Crespo‐Bojorque and Toro [45] found that changes in a sequence of consonant intervals are rapidly processed independently of an individual's musical expertise, as revealed by a change‐related mismatch negativity (MMN) elicited in both musicians and nonmusicians. In contrast, changes in a sequence of dissonant intervals elicited a late MMN only in those participants with prolonged musical training. Furthermore, magnetoencephalography studies by Tabas et al. [46] and Andermann et al. [47] further demonstrated that dissonant dyads evoke pitch onset responses with latencies up to 36 ms longer than consonant dyads, confirming that the auditory system takes longer to parse dissonant sounds.

Findings from nonhuman primates provide further evidence for the neurobiological grounding of different processing of consonance versus dissonance. Kadia and Wang [48] demonstrated that neurons in the monkey primary auditory cortex are selectively responsive to tones with fundamental frequencies and their harmonically related multiples. When presented with two tones, these neurons exhibit facilitation or inhibition that is enhanced when the tones share simple harmonic relationships. A couple of years later, Bendor and Wang [49] demonstrated that neurons in belt areas adjacent to primary auditory cortex respond not only to isolated fundamentals but also to their implied harmonic series, even in the absence of the fundamental itself.

Additional evidence suggests that consonance influences broader cognitive processes, including learning and executive function. For example, Crespo‐Bojorque and Toro [45] found that participants learned an abstract rule more effectively when it was implemented over consonant rather than dissonant intervals, indicating that consonance may facilitate pattern abstraction. Similarly, using a modified Stroop interference task (i.e., the color–word matching Stroop task [50]), Masataka and Perlovsky [51] demonstrated that exposure to consonant music (specifically, a Mozart minuet) reduced cognitive interference, whereas an altered version of the same piece with dissonant harmonies increased interference. This effect was observed in both children (8–9 years old) and older adults (65–75 years old), suggesting that the cognitive benefits of consonant music may be robust across age groups.

Throughout the centuries, theorizing on consonance has softened, if not revisited, the Pythagorean assumption that consonance can be fully explained in terms of simple integer ratios by considering a broader set of perceptual and neurophysiological factors, including interference among simultaneous partials, spectral harmonicity, and cultural exposure [5, 6, 10, 13, 52, 53, 54, 55].

One line of evidence challenging a strict identification of consonance with precise integer ratios comes from tuning studies comparing different tuning systems, such as Pythagorean tuning and equal temperament [56, 57, 58, 59]. Perceptual judgments of consonance have been shown to be unaffected by deviations from exact integer ratios, suggesting that consonance cannot be fully explained by ratio alone. Converging neurophysiological evidence supports this conclusion, with studies showing that neural responses evoked by equal‐tempered intervals closely resemble those elicited by Pythagorean intervals [60, 61, 62].

A further line of evidence underscores the importance of distinguishing perceptual sensitivity/discrimination from aesthetic or hedonic preference. In a cross‐cultural study, McDermott et al. [10] showed that members of an indigenous Amazonian population (the Tsimané) did not exhibit a preference for consonance in aesthetic judgments, despite showing an aversion to roughness and an ability to discriminate harmonic from inharmonic frequencies. This dissociation suggests that perceptual mechanisms supporting the detection and categorization of consonance and dissonance do not, by themselves, determine aesthetic preferences for consonance, particularly when such preferences are not reinforced within a given musical culture.

Taken together, the literature suggests that the ability to discriminate consonance from dissonance reflects early, possibly preattentive, stages of auditory information processing. These mechanisms operate at both subcortical and cortical levels and appear to reflect a broader principle of neural sensitivity to harmonic structure, with dissonant sounds requiring more computational effort than consonant ones. Their presence in nonmusicians and nonhuman primates suggests that consonance–dissonance discrimination may be a cross‐species and at least partially culture‐independent feature of auditory cognition.

While consonance perception cannot be reduced to sensitivity to simple integer ratios alone, it appears to emerge from the interaction of several interrelated psychoacoustic factors—such as harmonicity, beating, and roughness—that are themselves influenced by the frequency relationships between simultaneously presented tones. In particular, beating occurs when two tones with nearby frequencies interfere, producing slow amplitude modulations. For instance, two tones at 440 and 442 Hz produce slow beating (2 Hz) that the listener perceives as periodic increases and decreases in loudness. Roughness arises when the frequency difference between tones increases such that amplitude modulations fall within a faster range (approximately 20–150 Hz).

Recent contributions from dynamical systems theory have shifted toward a neural account of the observed privilege of low‐order ratios in harmony perception. In a comprehensive review, Harding and colleagues [22] advance an integrative framework based on NRT, according to which the brain is particularly sensitive to the stable resonance structures associated with low‐order rational frequency relationships. Neural resonance offers a common explanatory framework for different perceptual and neural processes, including sensitivity to harmonic structure, beating, roughness, and frequency relationships. Within NRT, harmonicity is not viewed as a competing explanation of consonance, but rather as a perceptual state arising from the stability of neural resonance. Similarly, beating and roughness are conceived as perceptual consequences of nonlinear cochlear and neural resonance dynamics [63]. The traditional opposition between ratio‐based, roughness‐based, and harmonicity‐based explanations is, therefore, reframed in NRT as reflecting different levels of the same description based on the brain's sensitivity to stable resonance structures associated with low‐order rational relationships.

## 3 Rhythm, Polyrhythms, and Integer Ratios

Based on the interstimulus intervals between successive pulses, rhythmic patterns can be categorized as either isochronous or nonisochronous. An isochronous rhythm occurs when each pulse is evenly spaced in time, resulting in a steady, regular beat. In contrast, a nonisochronous rhythm arises when the intervals between pulses vary, producing an irregular temporal pattern [64]. Additionally, more complex rhythmic structure arises in the form of polyrhythms, which involve the simultaneous presentation of two or more isochronous pulse trains whose periodicities align at points determined by the least common multiple of their respective cycles. In this section, we will briefly review the key evidence on rhythm and polyrhythm perception in humans, both in terms of basic discrimination ability and sensorimotor synchronization, with special attention given to the role of integer ratios in these abilities.

A terminological clarification is needed regarding the different ways ratios are used in the literature on rhythm and polyrhythms (see Table 1, see also the recent review by Nijhuis and colleagues [65]). In the case of rhythms, a ratio X:Y(:Z) refers to the (proportional) duration of consecutive sound events that form a rhythmic pattern. For example, a 1:1 rhythm consists of two beats of equal lengths. In a 1:1:2 rhythm, a bar is composed of three beats, the first two equally spaced and the third twice as long (e.g., a *Jingle Bells*‐like structure). In polyrhythms, the N:M ratio refers not to successive temporal intervals but to the frequency relationship between simultaneously occurring pulse trains, thus indicating that, for the case of 3:2 polyrhythm, a three‐beat rhythm is superimposed on a two‐beat rhythm within the same temporal cycle. When the onsets from the two streams are combined into a single rhythmic pattern, the resulting composite rhythm contains four onset positions per cycle, with successive onsets separated by temporal intervals in the ratio 2:1:1:2.

**TABLE 1:** The different meanings of ratios in the context of harmony, rhythm, and polyrhythms.

Context	Meaning	Example 3:2
Harmony	Superposition of two tones whose frequency ratio can be expressed as N:M	660 Hz:440 Hz, perfect fifth
Rhythm	A sequence of auditory events where the time intervals between successive pulses follow an X:Y(:Z) ratio	IOIs of 300 and 200 ms
Polyrhythm	Two concurrent pulse trains with different periodicities in an N:M ratio. When their onsets are combined, they form a composite rhythm with interval ratios of I:J(:K)	3‐beat stream superimposed to a 2‐beat stream. The resulting composite rhythm contains four onsets per cycle and an interval ratio of 2:1:1:2

Basic rhythmic skills have been demonstrated at a very early stage of development. For example, Demany et al. [66] demonstrated that infants (1.5–3 months of age) differentiate between a continuous sequence of identical 40‐ms auditory stimuli separated by 194‐ms intervals (i.e., 194–194‐194–194 ms), and another sequence of the same sounds having the identical duration with different spacing (i.e., 194–97–194–291 ms). The ability to discriminate between more complex rhythmic structures is evident in 5‐month‐old [67] and in 7‐month‐old infants [68], with infants showing early entrainment to simple meter rhythms [69].

Critical psychophysical and behavioral studies on adults indicate that rhythmic sequences built from simple integer ratios (such as 1:1 or 2:1) are perceived as more regular and easier to reproduce than those based on complex or irrational intervals [17, 19, 20, 21]. For instance, Essens and Povel [17] and Essens [70] found that rhythms with binary (1:2) or nonbinary integer (1:3) ratios were better reproduced than metrical rhythms with noninteger ratios (1:2.5 or 1:3.5; see also Sakai et al. [21]; Wu et al. [71]). When listening to isochronous rhythms with different tempi, adults readily differentiate them on the basis of their tempo, but performance declines significantly when adults listen to nonisochronous rhythms [72].

When asked to reproduce rhythms through clapping or tapping, participants show greater accuracy and consistency for integer‐ratio patterns. Spontaneous synchronization to external auditory rhythms has been demonstrated to occur at 1:1 and 1:2 ratios [73]. In studies of sensorimotor synchronization, findings converge, suggesting that the 1:2 ratio may be more accurately reproduced than the 1:3 ratio [18, 74]. In a cross‐cultural experiment with musicians, Polak et al. [19] tested synchronization with simple periodic rhythms at two different tempi with participants in Mali, Bulgaria, and Germany. The results provide support both for the assumption that 1:1 and 2:1 prototypes (often labeled as rhythmic priors in other studies [75, 76]) are widespread across cultures, with more complex ratios such as 3:2 and 4:3 being more culture‐dependent.

Critical evidence from Jacoby and McDermott [75] and a subsequent study by Jacoby and colleagues [76] demonstrates that listeners’ internal rhythmic priors consistently converge on simple integer ratios, regardless of musical training. However, these priors are shaped by cultural context. In the earlier study, both US participants and members of an Amazonian society exhibited preferences for integer‐ratio rhythms, but the specific ratios varied across cultures. Building on this, the 2024 study examined rhythmic priors across participants from 15 countries, revealing that while simple integer ratios are universally privileged, the prominence of particular ratios differs between groups, often reflecting local musical traditions. Together, such findings suggest that biological predispositions favor simple ratios, but cultural experience modulates how they are expressed in music perception.

Neuroimaging evidence suggests that the neural representations of rhythm are affected by ratios. For instance, using functional magnetic resonance imaging (fMRI), Sakai et al. [21] were able to show that rhythms constructed with small‐integer ratios (e.g., 1:2:4 and 1:2:3) activated similar brain regions (left premotor, parietal areas, right cerebellum), while more complex ratios (e.g., 1:2.5:3.5) recruited different, more widely distributed networks (right prefrontal, bilateral cerebellar posterior lobes). Similar results have been reported in comparative studies involving nonhuman primates. Macaques, for example, show neural sensitivity to structured rhythmic patterns based on simple ratios. Neurophysiological studies have demonstrated enhanced auditory cortex responses to 1:1:2 sequences, particularly at the position of the longer (accented) tone, suggesting an ability to track temporal accents aligned with metrical structure [77]. In addition, oscillatory activity in the delta band appears to align with the periodicity of such patterns, indicating that macaques—though not capable of full human‐like beat induction [78]—nonetheless display a preferential entrainment to regular, low‐complexity temporal structures. These findings support the notion that the sensitivity to simple ratios is not uniquely human but may reflect a more general auditory processing bias shared across primate species.

Rhythm perception and reproduction skills might also vary depending on individual exposure and musical background. Western listeners, typically exposed to isochronous and binary meters, show difficulty processing unfamiliar meters such as 3:2 or 5:3, especially in reproduction and synchronization tasks [70, 79]. Conversely, individuals from Turkey and India—where nonisochronous meters are more common—exhibit no bias toward simple binary ratios [80].

In the domain of polyrhythm perception, complexity emerges as a key factor influencing both perceptual processing and motor synchronization. Although not universally defined, complexity is closely linked to the polyrhythm ratio, with lower integer ratios such as 2:3 or 3:4 generally perceived as less complex than ratios like 5:6 or 7:8 [65, 81]. Due to the lack of shared metrics or indexes, motor responses, such as sensorimotor synchronization and coordination tasks, are frequently used in behavioral protocols as indirect measures of perceived complexity, with higher tapping accuracy or faster alignment to the beat indicating lower complexity [82]. For example, the study by Stupacher and colleagues [83] demonstrated that participants synchronize their tapping more quickly to a 2:3 than a 3:4 polyrhythm. Razdan and Patel [84] further suggest that the subjective pleasantness of polyrhythms negatively correlates with their mathematical complexity, a notion supported by numerical indices of difficulty proposed by Jagacinski et al. [85]. However, individual differences also play a role, with factors such as preferred motor tempo, musical training, and instrument familiarity modulating polyrhythm perception [83, 86].

## 4 Interim Summary

The reviewed evidence suggests that low‐order integer ratios are often associated with enhanced perceptual performance across both harmonic and rhythmic domains. These effects span multiple dimensions of auditory experience, including processing fluency, discrimination accuracy, and subjective clarity, thus lending support to the idea that the perceptual system may show increased sensitivity to periodic structures approximating low‐integer relationships.

### 4.1 Perceptual Fluency

In the literature on harmony perception, neurophysiological evidence shows that the auditory cortex requires more time to accomplish the early processing of dissonant versus consonant dyads [46, 47]. Consonant intervals generate waveforms with high temporal regularity, facilitating phase alignment across harmonics and promoting efficient neural encoding [35]. This temporal alignment is thought to contribute to perceptual fusion, one of the bases of consonance perception [87, 88].

Relatedly, evidence showing increased activity in the gamma band in response to consonant versus dissonant stimuli [9, 89] might be interpreted in light of the perceptual literature showing that gamma band activation is associated with more coherent percepts in vision [90, 91], thus confirming that dissonant sounds might produce a breakdown of the percept of a unitary sound. This hypothesis receives further support from a study considering object‐related negativity (ORN), an electrophysiological measure related to the concept of holistic grouping of stimuli that is allegedly related to coherence. In particular, Itoh and colleagues [92] showed that the minor second elicited significantly greater ORN than the perfect fifth at the P2 latency (160–180 ms). A recent study shows that inharmonic sounds with a constant jittering pattern generate ORN and are more likely to be perceived as more than one auditory object [93]. Furthermore, frequency‐following response studies indicate that the perception of consonance versus dissonance might correspond to the fluency of neural processing for pitch at the level of the brainstem [36].

A parallel effect emerges in the rhythmic domain. Patterns built on simple ratios such as 1:1, 2:1, or 3:2 are more rapidly detected, learned, and reproduced than those involving irrational or complex integer ratios [17, 21]. Even rhythmic patterns built on a noninteger ratio that is close to 1:2 may be reproduced less accurately than a ternary subdivision ratio (3:1) [94]. This advantage is likely due to the predictable temporal structure of such rhythms, which aligns more readily with internal timekeeping mechanisms and neural oscillatory entrainment.

### 4.2 Discrimination Accuracy

The reviewed literature showed that simple ratios are associated with faster processing and enhanced sensitivity to deviations across harmony and rhythm. In harmonic perception, just‐noticeable‐difference thresholds for mistuning are lower for consonant intervals, indicating finer sensitivity to alterations in well‐structured frequency relationships [87, 95]. Moreover, lower‐order integer relationships have been associated with enhanced memory performance in melodic pitch perception tasks [30, 31, 32]. In the rhythmic domain, similar results emerge, with listeners detecting phase shifts or timing perturbations more accurately when they occur within polyrhythms based on simple ratios (e.g., 2:3, 3:4) than within more complex configurations (e.g., 5:4, 7:6) [83, 96, 97]. These findings suggest that perceptual systems are optimized for detecting low‐complexity temporal patterns.

### 4.3 Subjective Clarity and Stability

In addition to objective measures, subjective ratings consistently favor simple‐ratio stimuli in terms of clarity, stability, and aesthetic appeal. Consonant intervals are typically judged as smoother and more stable than dissonant ones [6, 28]. Similarly, polyrhythms based on low‐integer ratios (e.g., 2:3) are more easily tapped along to than those created on complex ones (e.g., 3:4), which might reflect increased predictability and easier encoding [83, 98].

Cross‐cultural studies suggest that, while the specific (preferred) intervals or rhythms preferred may vary with musical exposure [80], the underlying sensitivity to periodicity and simplicity is shared across populations [75]. This points to an auditory bias toward structured, low‐complexity stimuli, refined by cultural learning.

Simple integer ratios also influence psychoacoustic features such as auditory fusion—the tendency to hear multiple frequencies as a single percept [95, 99, 100, 101]—and roughness, a low‐level acoustic correlate of dissonance [102, 103, 104]. Consonant intervals, which align harmonically, are associated with decreased roughness and are typically perceived as more pleasant [88]. Likewise, fusion is enhanced when frequency components exhibit regular alignment, reinforcing the perceptual salience of simple ratios [95].

The convergence of behavioral, neurophysiological, and psychoacoustic data across harmonic and rhythmic domains raises the question about the putative existence of shared constraints in neural encoding, attentional dynamics, and sensory‐motor integration. To address this question, we examine how concepts from dynamical systems theory, auditory neuroscience, and motor control converge on the idea that low‐order frequency relationships serve as attractors for perception, action, and prediction across harmony and rhythm.

## 5 Toward an Integrated Ratio‐Based Perception‐Action Framework

Referring to the compelling structural similarities between pitch and rhythm, Pressing [105] wrote in terms of cognitive isomorphism. An intuitive way to illustrate such isomorphism is to consider that both tones and rhythmic patterns are, at their core, periodic stimulation unfolding in time. For example, two tones that form a harmonic interval, such as a perfect fifth (e.g., 300 and 200 Hz), are related by a 3:2 frequency ratio, meaning that for every three cycles of the higher tone, two of the lower tone occur. If these frequencies are scaled down—for instance, to 3 and 2 Hz—the same ratio persists, but the percept shifts from pitch to rhythm and, in particular, to a 2:3 polyrhythm. The auditory system now interprets the stimuli as temporally distinct events rather than continuous tones. However, while both harmony and rhythm perception involve the processing of structured periodicity, the timescales of pitch and rhythm are obviously different. We, therefore, move on to explore the possibility that shared processing mechanisms could be used to explain the perception of stimuli in both temporal regimes.

NRT has been proposed as a comprehensive account for the processing of musical structures [22]. NRT proposes that musical perception and coordination emerge from the synchronization—or mode‐locking—of neural oscillators with external rhythmic and tonal stimuli [106, 107]. Unlike predictive coding models, which rely on top‐down prediction error minimization, NRT suggests that perception arises from physical resonance patterns shaped by the dynamical stability of neural systems. Importantly, NRT does not posit explicit computation of ratios. Rather, as will become clear, low‐order ratios mark regions where coupled oscillators exhibit robust mode‐locking. These regions form stable attractor states in nonlinear dynamical systems that span auditory and motor networks and are refined over time through Hebbian learning, a process known as *attunement* [108]. In this way, NRT can account for oscillatory phenomena from slow rhythms to high‐frequency pitch structures, such as pulse perception, meter, and consonance, by linking them to the stability of coupled oscillations across timescales.

Key concepts from nonlinear dynamical systems theory have often been evoked in NRT as a powerful framework for understanding why rhythmic coordination tends to stabilize around simple frequency ratios. According to this view, rhythmic patterns emerge from the interaction of coupled oscillators, which can represent neural circuits processing temporal structure [109]. Nonlinear neural oscillators can generate and synchronize to frequencies that are not physically present in the input but are related to it (e.g., missing fundamental) [110], a property observed throughout the auditory system from the cochlea and brainstem to auditory cortex [49, 111]. Through mode‐locking, oscillations with different natural frequencies stabilize when they form simple integer ratios, with stability increasing for simpler ratios and stronger coupling [108, 112]. In this framework, musical structure and expectancy arise because neural dynamics tend to move from less stable to more stable resonant states, constraining both perception and performance (e.g., simple rhythmic ratios are easier to coordinate than complex ones).

**FIGURE 1:**
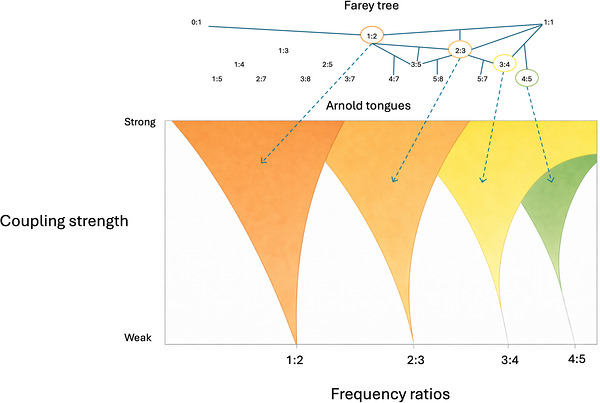
Schematic illustration of how low‐order integer relationships correspond to broader regions of stable phase locking in coupled oscillatory systems. Top: A portion of the Farey tree showing structural relations among ratios. Bottom: Schematic representation of Arnold tongues representing regions of dynamical stability in the plane of forcing amplitude F and frequency ratio. Each tongue corresponds to a k:m phase‐locking regime. In the weak‐coupling limit (F→0), locking occurs only at exact rational ratios, and the tongues collapse to points. As the forcing amplitude increases, the locking regions widen, indicating an increasing tolerance to frequency detuning. Low‐order integer ratios such as 2:1, 2:3, or 3:4 support stronger and more resilient neural synchronization. Higher‐order resonances (larger k+m) produce progressively narrower tongues. Ratios on the left branch of the Farey tree (1:3, 1:4, 1:5) are omitted for clarity, as they map under octave equivalence to the same perceptual intervals represented on the right branch (e.g., fifth, major third, octave).

In their influential work, Treffner and Turvey [113] demonstrated that when two oscillatory systems interact, they tend to synchronize in patterns governed by mathematical structures like the Farey sequence and the circle map. The Farey sequence is a series of irreducible fractions arranged in ascending order according to their numerical value, where each fraction represents a possible ratio between oscillators. For instance, the Farey sequence of order 5 is the sequence of irreducible fractions between 0 (or 0/1) and 1 (or 1/1) that have denominators less than or equal to 5, arranged in order of increasing size (see Figure 1). It is noteworthy that the Farey tree has been used to approximate the complexity of ratios across harmony, rhythm, and polyrhythm domains [65, 114, 115].

The circle map is a nonlinear dynamical model that describes how the phase relationship between two coupled oscillators evolves over time; it reveals how stable phase‐locking occurs at certain ratios depending on the strength of coupling and frequency detuning. Zeller et al. [116] demonstrate that the temporal dynamics of spiking neurons can be effectively approximated using one‐dimensional circle maps. Their study reveals that coupled neural assemblies can synchronize through weak interactions, with global behavior reducible to simple resonance principles. This work supports the claim that neural dynamics favor stable integer‐ratio entrainment patterns, offering evidence that the brain's processing of rhythmic and harmonic structure may stem from generic principles of nonlinear synchronization. Both the Farey tree and circle map predict that the most stable coordination modes occur at low‐order integer ratios (e.g., 1:1, 1:2, 2:3), where one oscillator completes a fixed number of cycles relative to another.

The mode locks can be visualized as Arnold tongues, namely, stable resonance regions in a parameter space where specific ratios dominate (see Figure 1). The wider the tongue, the more robust the synchronization. These mode‐locked synchronization patterns have clear biological counterparts across systems. In the domain of neural entrainment, for instance, oscillations in auditory cortex align their phase with external rhythmic input, such as a beat or metrical pulse.

According to this view, neural populations naturally entrain—that is, align their phase and frequency—to the periodic structure of incoming stimuli across multiple temporal scales [117, 118, 119, 120, 121]. For example, listening to a metronomic beat induces slow neural oscillations and/or amplitude modulations in faster oscillatory bands that mirror the temporal regularities of the stimulus, including its multiples and subharmonics [48, 49]. Critically, the stability of these phase relationships depends on the ratio between oscillatory cycles. Low‐order integer ratios such as 1:1, 2:1, or 2:3 support stronger, more resilient synchronization, whereas more complex ratios are less stable [122, 123]. Shapira Lots and Stone [124] proposed that musical consonance emerges from mode‐locking in nonlinear oscillator networks, while related models have used stylized neural dynamics and neural network architectures to simulate the encoding of consonance and dissonance based on oscillator synchronization [125].

From this perspective, perceptual advantages associated with integer‐ratio stimuli may reflect underlying constraints on the brain's oscillatory architecture tuned to favor phase relationships that optimize coherence, efficiency, and stability [22]. Related models further propose that specific neuronal ensembles or channels are selectively responsive to particular durations or tempi [126, 127], reinforcing the idea that rhythm and timing perception is scaffolded by biological tuning to structured temporal regularity.

Neurophysiological evidence suggests shared or overlapping neural mechanisms between pitch and rhythm processing. For instance, cortical entrainment to periodic acoustic stimuli shows similar dynamics no matter whether the input is spectral (harmonic) or temporal (rhythmic), suggesting that the auditory system uses general‐purpose periodicity detectors [22, 122, 128, 129]. These mechanisms appear tuned to maximize efficiency by prioritizing structure based on temporal coherence and simplicity, a principle consistent with predictive coding theories of perception, where the brain minimizes error by modeling the most statistically regular aspects of sensory input [96, 130]. Within this framework, low‐order integer ratios might represent efficient, low‐complexity temporal structures that the auditory system preferentially encodes.

Behavioral and neurophysiological findings further demonstrate that noninteger ratios are often stretched or shrunk toward integer values during synchronization or reproduction [70, 75, 131, 132]. This rhythmic regularization might be related to the persistence of interval ratios closely approximating simple integer ratios across tuning systems despite slight deviations. For instance, just major thirds and Pythagorean counterparts correspond to frequency ratios of 5:4 (i.e., 1.25) and 81:64 (i.e., 1.2656), respectively. Taking a reference pitch of 440 Hz, these yield frequencies of approximately 552.6 and 550 Hz. The resulting difference of about 2.6 Hz (≈ 0.5%) lies near or below the discrimination threshold for most listeners in normal listening conditions, and surely in the tolerance range of auditory system [3], as suggested by studies showing that neural processing of intervals is influenced by the degree of consonance even in the case of equal temperament, despite the complex ratios that characterize it [37, 92]. This phenomenon likely occurs because resonance peaks on the basilar membrane are broad enough to allow the nervous system to accommodate small deviations while maintaining stable perceptual categories [133]. Such conclusions align with the idea that musical intervals, especially when sung, are best understood as regions of closely related frequencies that may partially overlap, rather than as exact integer ratios defined by discrete categorical boundaries [134]. In NRT, perceptual categories correspond to resonance regions rather than exact frequency relationships. The breadth of these regions depends on coupling strength and experience‐dependent attunement [22, 106, 108].

Closely related phenomena have recently been observed in the neural processing of rhythm. Barbero and colleagues [135] provide compelling evidence for categorical neural representations of rhythmic structure in humans. Participants listened to a continuum of rhythmic sequences defined by repeating patterns of two inter‐onset intervals, spanning ratios from 1:1 to 2:1. Analysis of neural responses revealed a categorical organization of rhythm, with two distinct neural clusters: a smaller category encompassing ratios approximately between 0.50 and 0.55 (corresponding to 1:1 to about 1.2:1), and a larger category spanning ratios from roughly 0.56 to 0.67 (approximately 1.3:1 to 2:1). Importantly, these neural rhythm categories emerged automatically, independent of overt motor production or explicit timing tasks, yet closely mirrored categorical boundaries observed in behavioral responses.

Importantly, NRT can also accommodate findings showing perceptual advantages—such as more stable memory traces—for low‐order relationships in sequential contexts, including melodic intervals [30, 31, 32, 33]. Large et al. [136] demonstrate that the greater dynamical stability associated with mode‐locking at simple ratios predicts perceptual stability within tonal hierarchies, and this logic extends naturally to sequential patterns. When successive tones stand in a simple relationship, the oscillatory traces generated by the events reinforce one another through stronger nonlinear resonance. When relationships are more complex, coupling is weaker, and traces decay more rapidly. Computational work by Kim [137] provides a more explicit implementation of this idea for pitch memory and sequential structure, showing how consonant leaps can yield longer‐lasting oscillatory traces via mode‐locking dynamics that then shape implied harmony and tonal organization. Finally, findings from a recent study by Demany and colleagues [138] supported that simple frequency ratios are intrinsically advantageous for the perceptual encoding of melodies.

It is noteworthy that the presence of simple ratios in harmonic intervals is not confined to Western musical conventions, with ethnomusicological studies showing that the main musical traditions in the world make specific use of many of the same intervals [134, 139] and that the most frequently used intervals correspond to those considered more consonant by culturally diverse listeners [134, 140]. Additionally, developmental research finds that infants display preferences for consonant intervals and regular rhythms before extensive musical exposure [69, 141, 142], suggesting that sensitivity to these ratios might be a species‐wide auditory trait rooted in biological constraints [143].

An intriguing question now arises: How do the perceptual and processing advantages associated with simple integer ratios extend to the motor domain? Only a few studies have provided insightful findings into the link between perceptual advantages and motor responses in the rhythmic domain. For instance, experiments [144] using unimanual and bimanual tasks, where competing frequencies were imposed implicitly to maintain a specific polyrhythm, demonstrated shifts in actual frequency ratios toward simpler, more stable ratios like 1:1 or 1:2, and these shifts conformed to the unimodular structure of the Farey tree. Additional support comes from a more recent electroencephalography study on auditory‐motor synchronization, which showed that neural measures of entrainment to the stimulus decreased as rhythmic complexity increased during tasks (1:1 vs. 3:2) [145].

Support for this embodied perspective comes from another domain governed by rhythmic coordination: LRC. While a wide range of frequency ratios is theoretically possible, empirical observations reveal a restricted and highly structured set of LRC patterns [146, 147]. Simple ratios such as 1:1 and 2:1 are the most frequently reported and are closely associated with specific locomotion modes. For example, a 1:1 coupling between stride and breathing cycles is commonly observed during walking in humans and quadrupeds [148, 149, 150]. A 2:1 ratio (two strides per respiratory cycle) emerges reliably during running in humans and during trotting in horses, reflecting biomechanical constraints imposed by limb impact and visceral dynamics, although ratios such as 4:1, 3:1, and 3:2 can be observed in specific phases [148]. Very high ratios (up to 10:1) have been reported in dogs at high locomotor frequencies [149]. In flying birds, the most common ratios of wingbeats to breaths are 1:1, 2:1, 3:1, 4:1, though some more complex ratios have also been reported (e.g., 7:2) [151]. More complex ratios, such as 5:4, seem absent, and are predicted to be unstable by models such as the sine circle map [112].

The prevalence of such ratios seems to reflect a coupling mechanism that maximizes energy efficiency and dynamic stability [152, 153]. Notably, humans also favor low‐integer LRC ratios, with performance accuracy declining as ratio complexity increases [148], and even when complex patterns are acquired (e.g., 5:3), they often reflect combinations of simpler, more stable components [153]. Supporting this, Laffi et al. [154, 155] show that equine gaits conform to characteristic timing ratios, suggesting that natural movement systems are organized around simple temporal regularities. Recent studies have also proposed mathematical approaches to the analysis of repetitive and cyclic movements, such as walking and swimming, based on generalized Fibonacci sequences of finite length—a concept strongly related to the Farey tree—by associating the durations of the subphases of the gesture to the elements of such sequences [156, 157].

Moving to the harmonic domain, Komeilipoor et al. [158] showed that movements synchronized either to consonant or to dissonant sounds were differentially influenced by the degree of consonance of the sound presented. Interestingly, the difference was present after the sound stimulus was removed, with motor performance after consonant sound exposure found to be more stable and accurate as compared to performance measured after exposure to dissonant sounds. More recently, Lazzari et al. [159] had nonmusician participants in dyads perform a dyadic synchronization‐continuation task: on each trial, participants first synchronized their movements to a metronome (synchronization phase) and then continued tapping together at the same tempo without the metronome (continuation phase). Each tap produced a musical tone, and participants heard both their own and that of their partner, thus creating an interval that varied from consonance to dissonance. The results showed that participants’ taps were closer in time when they jointly created consonant versus dissonant intervals.

Furthermore, Matthews and colleagues [160] demonstrated that the sensation of groove is influenced by both rhythmic and harmonic complexity. In their study, the authors created short musical sequences with three levels of rhythmic and harmonic complexity (low, medium, and high). Participants were asked to rate each stimulus in terms of pleasure and the urge to move. The results showed that stimuli with high harmonic and rhythmic complexity were rated as less pleasant and elicited a lower urge to move, whereas those with medium and low complexity were rated at the highest and intermediate levels, respectively (see also the study by Spiech et al. [81]). The authors of the study propose that harmony modulates the perceived affective quality of music and, in doing so, alters the degree of entrainment to rhythmic stimuli. This modulation may, in turn, affect the precision of temporal prediction processes that depend on such entrainment. Converging evidence comes from a study by Fritz and colleagues [161] involving patients with Parkinson's disease, in which consonant and dissonant musical pieces were presented while gait parameters were recorded. The results showed superior gait performance, namely, higher walking velocity and longer stride length, when participants listened to consonant music compared to dissonant counterparts.

These findings suggest that the neural resonance triggered by the perception of simple integer ratios—in both rhythmic and harmonic domains—leads to enhanced motor synchronization. This paves the way for linking perceptual and processing advantages to motor outputs, grounding musical behavior in sensorimotor coupling. Here, embodied music cognition theory offers a compelling interpretive lens: it posits that musical experience is not merely auditory, but fundamentally rooted in bodily action and sensorimotor engagement with sound.

As proposed by Leman [162], musical meaning emerges through action‐oriented interactions between listener and environment, with the body acting as a mediator that maps auditory input onto motor affordances. Building on this, Maes et al. [163] argue that perception and action are dynamically coupled processes in musical experience, shaped by the brain's predictive use of motor simulation to process rhythmic input. From this perspective, the stability and predictability afforded by simple integer ratios in rhythm may foster efficient motor simulation and resonance, facilitating entrainment, movement coordination, and even social bonding through shared rhythmic structures.

**FIGURE 2:**
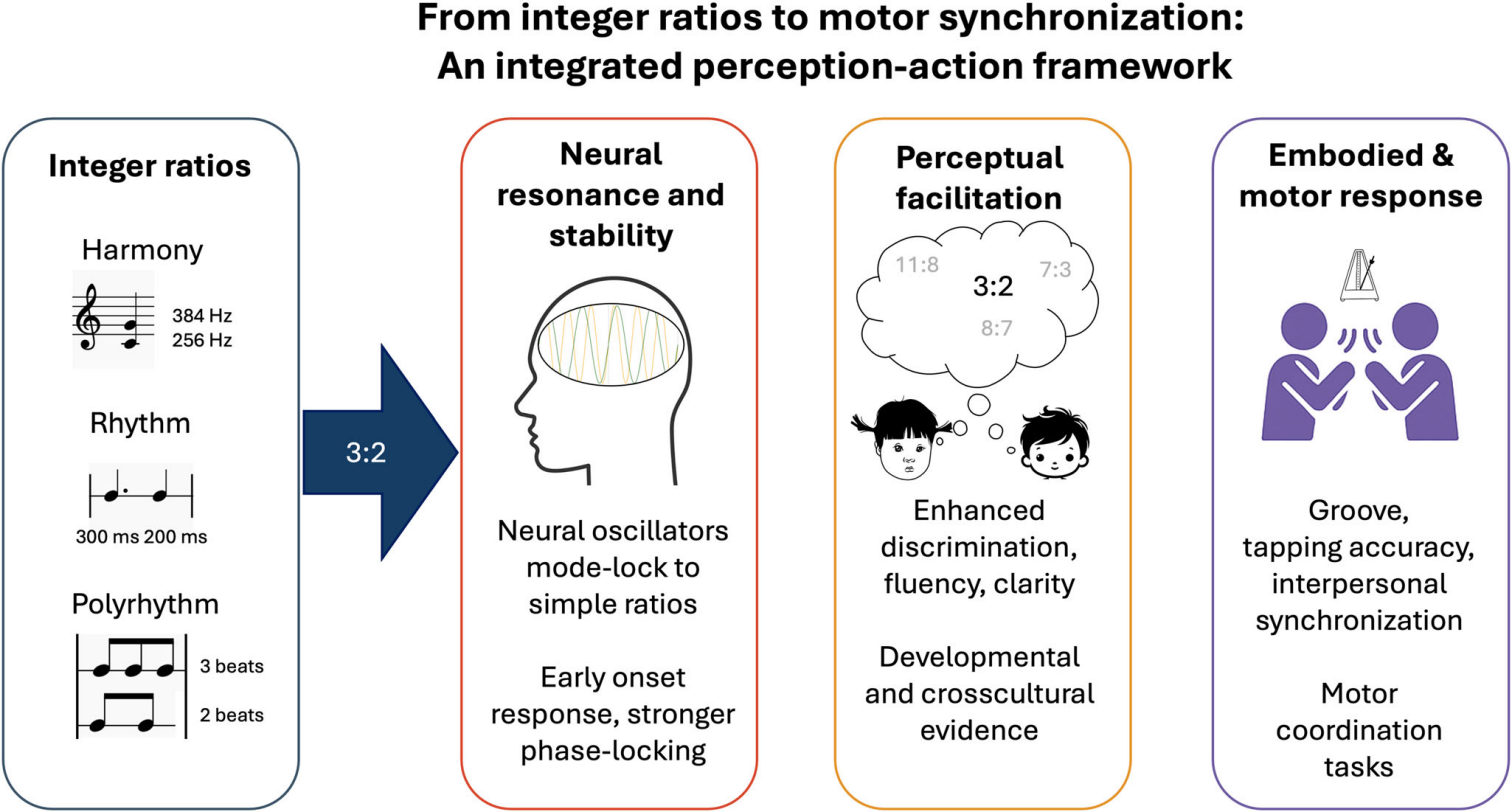
Illustration of how simple integer ratios in harmony and rhythm propagate through neural dynamics, affecting perceptual and motor responses.

These findings resonate with broader theories of embodied cognition [164, 165], which posit that perception, cognition, and action share common representational formats and neural substrates. Thus, the stability of simple ratios in biological rhythms lends support to the idea that musical meter and harmony may draw upon deeply rooted principles of bodily coordination and adaptive control (see Figure 2).

## 6 Conclusions and Future Directions

The present framework proposes that low‐complexity frequency relationships are privileged across harmony and rhythm because they correspond to stable coordination regimes that emerge naturally in coupled dynamical systems, including neural and sensorimotor systems, that underlie pitch and rhythm perception. From this perspective, perceptual and motor advantages for low‐order relationships reflect broad resonance regions in which phase locking is robust to variability, with tolerance shaped by coupling strength, experience, and context. Differences across tuning systems, mistuning tolerance, and cultural exposure are, therefore, coherent consequences of operation within flexible stability regions rather than at precise numerical ratios. NRT provides a unitary theoretical framework by explaining how stimulus properties (i.e., simple integer ratios) give rise to specific neural responses (e.g., stronger mode‐locking, more stable neural dynamics, and more coherent oscillatory activity), which in turn may underpin behavioral advantages such as improved memory, discrimination, and motor synchronization.

Several intriguing questions remain open and require targeted empirical work. One important direction involves systematically manipulating ratio complexity along a continuous scale. Most existing studies contrast canonical simple ratios (e.g., 2:1, 3:2) with irregular or complex ones, but few chart the full psychometric function that links ratio simplicity with perceptual fluency, discrimination, or subjective preference (see [132] for an exception). Stimuli parametrized by numerical complexity—such as Farey sequences or continued fractions—could be used to clarify whether perceptual responses vary linearly, categorically, or logarithmically with ratio structure. Additionally, to disentangle between event density and ratio complexity in polyrhythms perception, future studies that equate the number of presentation cycles rather than duration across conditions would provide a useful test of whether low‐order relationships retain a stability advantage under controlled event density and repetitions of the pattern.

While cortical entrainment and oscillatory coupling have been observed in both harmonic and rhythmic domains, more targeted studies using comparable stimuli and tasks across timescales would help determine whether analogous periodicity‐tracking mechanisms operate across systems. Combining neurophysiological data from multiple methodologies with predictive processing and dynamical‐systems approaches may clarify whether low‐order frequency relationships correspond to stable coordination regimes at both neural and behavioral levels, without assuming explicit neural computation of numerical ratios. Furthermore, future studies could directly compare perceptual tolerance for low‐order relationships in harmony and rhythm, helping determine whether similar computational constraints shape processing across these domains. Noninvasive brain stimulation techniques (e.g., transcranial magnetic stimulation) could be used to test the causal contribution of candidate regions—such as auditory and motor areas—to sensitivity to harmonic and rhythmic structure, while acknowledging that different regions are likely to contribute at different temporal scales.

Similar to what has been demonstrated by Crespo‐Bojorque and Toro [45] in the domain of harmony, future research could systematically investigate whether rhythmic structures based on simple integer ratios modulate cognitive and executive functions. For example, such studies might examine the extent to which temporal regularities influence attentional control, working memory, or cognitive flexibility across different cultural contexts. A key unanswered question concerns the developmental emergence of polyrhythm perception. Future work should, therefore, assess whether infants and young children display an early preferences for simple rhythmic ratios. Protocols could also be designed to test the effect of prenatal exposure to polyrhythm perception on infants’ brain/behavioral responses [166]. Such findings could reveal whether the salience of low‐order ratios in rhythm, like in harmony, emerges independently of formal musical training.

Finally, future research should investigate whether the perceptual and processing advantages associated with integer ratios in auditory perception extend to other sensory modalities, such as vision and touch. While the empirical evidence published to date in this domain remains sparse and inconclusive [167, 168], research on multisensory integration and crossmodal correspondences suggests that shared structural principles may govern perceptual organization across the senses [169, 170, 171]. Within this perspective, sensitivity to simple temporal ratios may not be specific to audition, but instead reflect more general constraints on how the brain encodes and integrates temporal information across sensory systems.

## Author Contributions

N.D.S.: Conceptualization; writing – original draft; writing – review and editing; funding acquisition; E.L.: Writing – review and editing; supervision; C.S.: Conceptualization; writing – original draft; writing – review and editing; supervision.

## Conflicts of Interest

The authors declare no conflicts of interest.
